# Subtle ocular motor deficits in people with chronic whiplash associated disorder compared to healthy controls

**DOI:** 10.3389/fneur.2025.1676654

**Published:** 2025-10-16

**Authors:** Brad Callan, Antonio Vintimilla, Nicholas Gulla

**Affiliations:** ^1^School of Physical Therapy, Pacific Northwest University of Health Sciences, Yakima, WA, United States; ^2^School of Physical Theraphy, Wexner Medical Center, The Ohio State University, Columbus, OH, United States

**Keywords:** whiplash, concussion, post-concussive syndrome (PCS), ocular, cognitive, balance

## Abstract

**Introduction:**

More than 50% of people who are diagnosed with whiplash-associated disorder (WAD) will report symptoms 12 months and beyond after their initial onset. However, many signs and symptoms, such as dizziness, emotional lability, confusion, ocular movement abnormalities, and balance deficits, may not be directly attributed to the cervical spine and may be more consistent with post-concussive syndrome (PCS).

**Methods:**

A total of 15 people with chronic (>3 months) WAD and 15 age-sex match controls were recruited. They were evaluated on clinical tools commonly used to assess signs and symptoms associated with concussion and PCS, including self-report symptoms, balance, cognition, and vestibular-ocular assessments. All scores were assessed for differences between the two groups, and effect sizes were recorded.

**Results:**

All testing, except for balance, demonstrated significant differences between the groups. Within the ocular motion, 31/34 variables moved less efficiently in the WAD group. Using an exact binomial paired sign test, the likelihood of all eight ocular composite groups being less efficient in the WAD group is reported as *p* = 0.008.

**Discussion:**

Patients with chronic WAD demonstrate subtle but significant differences in ocular movement when compared to a control group. They also demonstrated significant differences on measures commonly used in the assessment of PCS despite never being diagnosed with it. These differences may contribute to some of the ongoing disability burden that this population commonly reports.

## 1 Introduction

Whiplash-associated disorder (WAD) is recognized as a constellation of signs and symptoms, including but not limited to neck pain, body pain, dizziness, fatigue, cognitive deficits, and balance disturbances (1). These often follow traumatic incidents, the most common being a motor vehicle collision (MVC). Traumatic eventa expose the head and neck to rapid acceleration-deceleration forces, frequently leading to cervical spine injuries and, in some cases, traumatic brain injuries (TBIs) (2, 3).

A TBI can occur from direct cranial impact or from sudden movement of the brain within the skull (4). Moderate and severe TBIs are typically easy to recognize following an MVC due to their critical presentation. However, a mild TBI (mTBI), commonly referred to as a concussion, usually presents with more subtle symptoms such as dizziness, fatigue, cognitive deficits, balance disturbances, and ocular dysfunction, making it difficult to diagnose (4). Limited reliable objective measures for concussive further complicate injury management. Although symptoms often resolve within a month, persistence beyond that period may lead to a diagnosis of post-concussive syndrome (PCS) (5). In some cases, symptoms can persist for months or even years following the initial injury (6).

The signs and symptoms of chronic WAD and PCS overlap considerably, making it difficult to differentiate between them (1, 2). However, following an MVC, the clinical focus tends to prioritize neck pain, while often overlooking the potential for a concussion and possible PCS as a driver of symptoms (7–9). Given the forces experienced in an MVC, concussions and subsequent PCS may be underreported.

Several studies examining people with WAD have explored variables that are associated with concussions, such as balance, cognition, vestibular function, and ocular motion (10–13). Although changes in many of the systems have been observed, ocular movement assessments have been less robust (14, 15). One limiting factor may be that ocular examinations have historically relied on manual assessment techniques to assess movement. While this method may be appropriate for individuals with a moderate or severe TBI, it may not detect the more subtle changes seen in other cases (16). Using an objective, standardized tool, such as the RightEye® vision system, to examine ocular motion in individuals with WAD could provide more definitive results in this area.

The purpose of this article is to evaluate individuals with chronic WAD using clinical tools commonly employed to assess patients with PCS, and to compare their results to those of a healthy, matched control group. We hypothesize that individuals with chronic WAD will demonstrate differences from the control group on assessments of PCS-related functions, including balance, cognition, and ocular movement.

## 2 Methods

### 2.1 Data collection

Data were collected from a cross-sectional convenience sample of participants. People were recruited from the local community through word-of-mouth, social media, and personal communications. The inclusion criteria were: age between 18 and 60 years, a diagnosis of grade 2–3 WAD on the Quebec Task Force (QTF) scale following a MVC at least 3 months prior, and fluency in English or Spanish. The exclusion criteria included a previous diagnosis of TBI or concussion; a history of nervous system disorders (such as seizures, epilepsy, multiple sclerosis, peripheral neuropathy, stroke, or Parkinson’s); diabetes; vision disorders other than the use of glasses or contacts; pregnancy; cervical spine surgery; whiplash classified as QTF grade 4; or prior treatment with or exposure to the RightEye® vision system. The control group was recruited as an age-sex matched cohort with no history of WAD or concussion.

### 2.2 Sample size estimate

Using GPower, an *a priori* sample size was calculated for matched pairs t-tests. Effect sizes were calculated from two different studies that assessed the RightEye® system in individuals with concussion (17, 18). Reported effect sizes ranged from 1.45 for larger measures, while sub-measures were lower, 0.42 and 0.57. Most of the metrics used in the analysis are considered sub-measures. To maintain a conservative approach, a total effect size of 0.7 was chosen. Alpha was set at 0.05 with a power of 80%. A total set of matched pairs was calculated at 15, or a total sample size of 30 between the two groups.

### 2.3 Subjective outcome measures

PCS consists of many of the same signs and symptoms as an acute concussion. While there are a variety of tools to examine those with an acute concussion, there are no specific tools used to examine a person with PCS. Therefore, common tools used to assess acute concussion were used in this study.

The Neck Disability Index (NDI) and Rivermead Post Concussion Questionnaire (RPQ) were used to determine self-reported outcomes. The NDI, the most widely used outcome measure in WAD research due to its reliability and validity, is used to obtain subjective data related to impairments in a person’s daily activities due to neck pain and/or symptoms (19). Although not used as a diagnostic tool for concussion or PCS, the RPQ evaluates common symptoms reported by people following a suspected concussion and can be used to monitor symptoms over time. The RPQ is commonly used in clinical and research settings and has demonstrated adequate reliability and validity in people who report both acute and chronic symptoms (20). Although a total impairment score can be reported as a percentage for each measure, raw scores were used in the analysis.

### 2.4 Objective assessments

The objective assessments used in this study were among the most commonly used in concussion research and included the Balance Error Scoring System (BESS), the Standardized Assessment of Concussion (SAC), the Vestibular Ocular Motor Screen (VOMS), and the RightEye® dynamic vision test.

The BESS is a validated and reliable tool used to assess a person’s balance with their eyes closed on two different surfaces and in three positions, and it is frequently used in the assessment of concussions (21). The total number of errors is counted and combined to create a total score; thus, a higher score represents poor balance. Individuals with a concussion generally demonstrate a higher number of errors (22).

The SAC is a cognitive assessment that is “sensitive to the effects of mild brain injury and concussion” (23). The SAC consists of four sections: orientation, immediate and delayed memory recall, and concentration. The highest possible score is 50, with lower scores indicating decreased cognitive abilities. Although the sensitivity of the SAC diminishes beyond 2 weeks, the authors are unaware of other readily available tools for cognitive assessment in this chronic population (24).

The VOMS is used to screen both vestibular and ocular movement in relation to symptoms of eye and head movements following a suspected concussion. It is considered a reliable and valid test in the screening assessment of a concussion (25). The test scores are based on a person’s symptoms in four distinct categories: dizziness, nausea, headaches, and fogginess after each motion. The symptoms are scored on a 0–10 scale. The motions include horizontal and vertical smooth pursuit, horizontal and vertical saccades, near point of convergence (NPC), horizontal and vertical vestibular ocular reflex, and visual motion sensitivity. With four symptoms and seven activities (symptoms were not recorded for near point convergence due to an error in training), the highest score possible is 280, and this number represents the maximal symptom burden.

The RightEye® vision system is a vision tracking system that uses infrared pupil tracking technology to objectively measure eye movements, including fixation, saccades, and smooth pursuits. The dynamic vision test tracks binocular eye movements during eight distinct activities: horizontal, vertical, and circular smooth pursuits; horizontal and vertical saccadic motion; fixation; and choice and discriminate reaction times. The RightEye® system provides objective monocular measurements of speed, errors, target tracking, and reaction times to stimuli for both the right and left eyes, as well as averages of the two. The RightEye® system has been reported to be a valid and reliable tool (26). It has been used in people with concussion and has demonstrated statistically significant differences when compared to healthy controls. The RightEye® collects data on numerous variables, but for this study, evaluation was based on 34 variables previously reported as being associated with concussion (18).

### 2.5 Statistical analysis

Statistical analysis was performed using SPSS v29 (Chicago, IL, United States). Baseline demographic information was collected and reported as the percentage of female participants, median age, and length of time since their MVC. For BESS, SAC, VOMS, and individual RightEye® variables, differences were determined using a paired t-test for ratio level data, a Wilcoxon signed-rank test for ordinal data, or data that did not meet the necessary assumptions. A separate analysis of the VOMS based on the NPC not being collected was examined for significance. The effect size r for each variable was calculated and categorized based on Cohen’s d, with 0.2 small, 0.5 medium, and ≥ 0.8 large (27). Due to the number of variables reported with the RightEye® and overlap between some of them (i.e., right and left eye measurements were reported separately), the variables were categorized into the eight distinct categories previously listed: horizontal, vertical, and circular smooth pursuit; horizontal and vertical saccades; fixation stability; and choice and discriminate reaction time. Within each category, individual variables were assessed via the difference in their mean score between the WAD and control groups. The total proportion of reported scores was listed, and an exact binomial paired sign test was used to determine the probability of the results favoring one group or the other.

This study was approved and overseen by the Pacific Northwest University Institutional Review Board (23–005).

## 3 Results

### 3.1 Baseline demographics

A total of 15 people with chronic WAD and 15 age-sex-matched controls were recruited. For demographic data related to the number of females: males, age, time since MVC, and median neck pain, see Table 1. Using a paired sample *t*-test, no significant difference in age between the groups was reported [t (14) = 1.75, *p* = 0.10]. Using a Wilcoxon signed-rank test for baseline neck pain, a significant difference was found (z = −2.966, *p* = 0.003). Using a chi-square test, no difference between the sexes and groups was observed, ꭕ^2^(1, *N* = 30) = 0.0, *p* = 1.0. The time since MVC was not evaluated between the two groups, given that the control group was not involved in an MVC.

**Table 1:** Demographics (sex, age, time since MVC, and average neck pain) for WAD and control groups.

Demographics	WAD group	Control group	Difference
Females: Males	13:2	13:2	N/A
Mean age in years (range)	35.9 (21–60)	34.3 (21–58)	*p* = 0.10
Median neck pain rated 0–10 (range)	2.0 (0–6)	0.0	*p* = 0.003
Mean time since MVC (in months)	75 (3–300)	N/A	N/A

MVC, motor vehicle collision; WAD, whiplash associated disorder.

### 3.2 Subjective outcome measures

For the NDI and RPQ, the raw scores were reported with median and interquartile ranges (IQR) listed in Table 2. As the NDI and RPQ are considered ordinal data, the Wilcoxon signed-rank test was used to determine differences. Assumption testing demonstrated a normal distribution around the mean. Because multiple tests were used to assess similar symptoms, a Bonferroni correction was applied (0.05/2), and alpha was adjusted to 0.025. A significant difference between the groups for both outcome measures was reported; for the NDI WAD median = 11.0 and control median = 0; z = −3.3, *p* < 0.001, and the RPQ WAD median = 27 and control median = 0; z = −3.4, *p* = <0.001. The effect size r was calculated for the NDI (*r* = 0.85) and RPQ (*r* = 0.88), both of which are considered large effects (27).

**Table 2:** Differences between the WAD and control group for NDI and RPQ.

Test	WAD (*n* = 15) Median; IQR	Control (*n* = 15) Median; IQR	Difference	Effect size r
NDI raw score	11; 8–20	0; 0–1	*p* < 0.001*	0.85
RPQ raw score	27; 16–33	0; 0–2	*p* < 0.001*	0.88

WAD, whiplash-associated disorder; IQR, interquartile range; NDI, neck disability index; RPQ, Rivermead post-concussion questionnaire. *statistically significant *p* < 0.05.

### 3.3 Objective measures

The BESS and SAC means and standard deviations (SDs), as well as the VOMS baseline and final score medians and IQR, are presented in Table 3.

**Table 3:** Differences between the WAD and control group for objective measures except RightEye®.

Test	WAD mean; SD (range)	Control mean; SD (range)	Difference	Effect size r
BESS	23.4; 8.1 (12–43)	19.8; 19.8 (9–33)	*p* = 0.06	0.41
SAC	36.0; 4 (31–47)	38.4 3.6 (31–44)	*p* = 0.05*	0.45
	WAD median; IQR	Control median; IQR		
VOMS baseline	3; 1–7	0; 0–0	*p* < 0.001*	0.83
VOMS final	17; 7–60	0; 0–5	*p* < 0.001*	0.85

WAD, whiplash-associated disorder; IQR, interquartile range; BESS, balance error scoring system; SAC, sports concussion assessment tool version 5; VOMS, vestibular ocular motor screen. *statistically significant *p* < 0.05.

BESS and SAC assumption testing for normality was deemed normal with the Shapiro–Wilk test, with significance being *p* = 0.167 and *p* = 0.369, respectively. To visualize the QQ plot, see Appendix 1. For the BESS, while the WAD group performed worse, a one-sided paired t-test found no significant difference between the groups; t (14) = 1.59, *p* = 0.067 (CI –1.25, 8.45). For the SAC, there was a significant difference between the two group means with the WAD group performing worse, t (14) = − 1.72, *p* = 0.050 (CI –5.39, 0.59). Effect size r was reported for BESS 0.41 and SAC 0.45, both of which are considered moderate effects.

Between the two groups, the VOMS baseline and total score demonstrated a non-normal distribution of the mean. However, this is not unexpected on the VOMS, given the clustering of scores in and around zero. With this distribution and the ordinal data from VOMS, a Wilcoxon signed-rank test was used. Given the test was twice (baseline and total), a Bonferroni correction was used (0.05/2), and the new alpha was set at 0.025. For the VOMS baseline, a significant difference between the distributions was found with the WAD group median = 3.0 and the control group median = 0; z = −3.20, *p* < 0.001. For the VOMS total score, a significant difference between the distributions was found with the WAD group median = 17 and the control group median = 0; z = −3.30, *p* < 0.001. These results indicate that the WAD group tended to have a higher score on the VOMS at both baseline and total score. A large effect size was calculated for both the VOMS baseline, *r* = 0.83, and the VOMS total score, *r* = 0.85.

Because NPC symptom data were not collected, an analysis was performed to determine whether the differences between the distributions would remain if NPC symptoms had been recorded. To create new scores for comparison for the WAD group, data were imputed from the vertical saccade scores, which were the assessment done immediately before NPC. This added an average of 4.9 units to each WAD participant. To ensure we were not missing any changes in the rank order, for the control group, 4.9 was doubled and rounded up to add 10 units to each participant. A Wilcoxon signed-rank test using these new VOMS numbers was run, and a significant difference between the distributions persisted, with WAD median = 19 and the control median = 10; z = −2.355, *p* = 0.019. Based on this *p-value* being below 0.025, we concluded that the missing NPC data had no bearing on the outcome.

For the RightEye® dynamic vision test, the variables examined used different metrics, including speed, accuracy, and variance, to name a few. To be succinct, the overarching theme of efficiency is used to describe the variety of results.

Assumptions of normality of the RightEye® data were examined using the Shapiro–Wilk test; nine variables were statistically significant (*p* < 0.05), indicating a non-normal distribution. All others were considered normally distributed. For those nine non-normally distributed variables, the Wilcoxon signed-rank test was used to determine group differences. A one-sided paired t-test was used for all other groups.

Of the 34 items examined, the control group was less efficient on two metrics (vertical saccades saccadic efficiency right and vertical saccades speed accuracy trade off right). One item was equal between the two groups (discriminate reaction time, visual reaction speed). For the other 31 items, the WAD group was less efficient. Effect sizes were calculated for each variable with a range from 0 to 0.652, with a mean of 0.294. Of the seven variables that had a significant difference between the two groups, the effect size r ranged from 0.529 to 0.652, with a mean of 0.56, which is considered moderate. See Appendix 2 for means, differences, *p*-values, and effect sizes of all 34 variables.

To determine overall differences between the groups, the vision testing variables were grouped into one of the eight categories corresponding to the RightEye® dynamic vision testing protocol. This protocol classifies outcomes based on the eight distinct oculomotor movements it evaluates, ensuring that related measures (e.g., right and left eye horizontal saccadic efficiency) are organized within the same movement domain. The categories are as follows: discriminate and choice reaction time; horizontal and vertical saccades; circular, vertical, and horizontal smooth pursuit; and fixation stability. The number of ocular variables within each category (range 2–8) and the more efficient group for each given variable category are listed in Table 4. Following these tallies, an exact binomial paired sign test was run to compare the likelihood of the WAD or control groups being more efficient within the categories. From the tallies, all eight categories favored the control group as being more efficient, and the difference was statistically significant with z = 2.475 and *p* = 0.008.

**Table 4:** Variable groupings and the number of variables that were deemed more efficient within each group.

Variable categories	WAD number of more efficient ocular movements	Control group number of more efficient ocular movements	WAD: Control ratio of efficient movements
Horizontal saccades	0	8	0:8
Vertical saccades	2	6	2:6
Circular smooth pursuit	0	2	0:2
Horizontal smooth pursuit	0	2	0:2
Vertical smooth pursuit	0	2	0:2
Fixation stability	0	3	0:3
Choice reaction time	0	4	0:4
Discriminate reaction time	0 (1 tie)	4	0:4 (1 tie)

WAD, whiplash-associated disorder.

## 4 Discussion

The objective of this study was to assess subjective, physical, and cognitive impairments in individuals with chronic WAD using clinical tests typically reserved for people with concussion and to compare them to a health-matched control group. Using a range of self-reported, physical, cognitive, and dynamic vision assessments, the study demonstrated significant differences across multiple domains, highlighting the complex, multifaceted nature of chronic WAD-related dysfunction.

Participants with chronic WAD exhibited significantly higher scores on both the NDI and the RPQ compared to controls. While higher scores are expected, there was a greater level of impairment on the RPQ (25.9/64 = 40.5% impairment) than on the NDI (12.7/50 = 25.3% impairment). This result is highlighted as the NDI is the most used self-report outcome tool in people with WAD, while the RPQ is used to assess symptom burden in people with a concussion. These findings indicate a pronounced degree of functional disability in individuals with chronic WAD. They also reinforce previous literature that suggests a strong overlap between cognitive and neurological symptoms between WAD and PCS (8, 28).

The physical and cognitive assessments revealed mixed results. The BESS demonstrated no significant differences between WAD and control. Previous research on postural sway and balance in individuals with acute and chronic WAD has generally reported significant differences (10, 13). However, in these studies, effect sizes were not reported and could not be calculated, so the authors cannot make specific judgments about these current results compared to prior studies. In this study, the *p*-value for BESS was 0.067 with a reported effect size of 0.411. However, the *a priori* sample size used a 0.7 effect size, and thus, a larger sample size may be necessary to recognize a true difference.

In contrast, a significant difference was observed in cognitive function, as assessed by the SAC. The WAD group demonstrated a significantly lower mean score (36.0) compared to controls (38.4). This suggests that chronic WAD is associated with mild but measurable cognitive deficits, particularly in memory recall and concentration, when compared to healthy controls. These results support a previous meta-analysis, which found cognitive changes in people who have had a whiplash injury and continue to have symptoms when compared to both healthy controls and non-symptomatic people who have also been in an MVC (29). However, in the previous analysis, they did not examine the SAC as we did here, which appears to strengthen the idea that cognitive impairments can manifest for extended periods in people following an MVC.

These cognitive impairments may reflect ongoing disruptions in central nervous system processing, consistent with central sensitization and neuroplastic changes frequently observed in people with chronic whiplash (30). In addition, two components of the dynamic vision test, choice reaction and discrimination reaction, are not explicitly cognitive tests but require a degree of central processing. In both categories, the WAD group was found to be slower and less accurate than the control group in 8/9 variables.

The VOMS revealed differences between groups, both at baseline and following dynamic movements. The baseline VOMS score for the WAD group, median = 3.0, was significantly higher than that of controls, 0.0. This indicates that even in a resting state, individuals with chronic WAD experience what can be considered vestibular and oculomotor symptoms. Furthermore, the total VOMS score after testing was elevated in both groups, WAD median = 17.0 compared to control median = 0.0, with a significant difference between them.

Vestibular and oculomotor dysfunctions may be prominent features of chronic WAD, potentially contributing to dizziness, nausea, headaches, and cognitive fog commonly reported in this population (28). In addition, alterations in the cervical-ocular reflex (COR) have been reported in people with WAD and non-specific neck pain (12). Given the role of the vestibulo-ocular reflex (VOR), COR, and their interactions in maintaining gaze stability and spatial orientation, deficits in these domains may have significant implications for daily activities, particularly those requiring dynamic visual and postural control (31, 32).

The dynamic vision test using the RightEye® system revealed that the control group moved more efficiently, faster, and more accurately, when compared with the chronic WAD group on 31/34 variables. While there was a significant difference between 7 of the 34 groups across all 34 variables, the average effect size of 0.294 indicates a small effect. However, there was an effect size of 0.56 across those variables that were significantly different. Like the BESS, these effect sizes are lower than the one used in the *a priori* sample size estimate (0.7); thus, a larger sample may have provided more groups with differences between them. Noting that, all eight categories showed a greater number of impairments in the WAD group, which may indicate widespread deficits in eye-tracking, reaction time, and fixation stability.

Previous research has reported that deficits in the vestibular systems, the COR, and even neck pain may influence ocular motion. However, during testing with the RightEye®, the head remains stationary, which eliminates or at least minimizes both the COR and VOR, as well as attempts to mitigate the influence of neck pain. The findings from this study demonstrate oculomotor dysfunction in people with chronic WAD compared to a control group, supporting the hypothesis of the potential central nervous system involvement in chronic WAD in addition to cervical spine dysfunction (33, 34). Combined dysfunction in the COR and VOR likely complicates these results beyond a single system. Further research into investigating how these systems interact will be necessary to unravel the complexity of WAD.

The results of this study have important clinical implications for the assessment and management of chronic WAD. First, the significant impairments observed across multiple domains underscore the need for a multidisciplinary approach to evaluation and treatment. Standard musculoskeletal assessments may fail to capture the full extent of functional deficits, particularly in cognitive and oculomotor domains. Incorporating tools such as SAC, VOMS, and dynamic vision testing may provide a more comprehensive understanding of the patient’s condition and guide targeted interventions. Second, the overlap in symptomatology between chronic WAD and PCS highlights the potential for misdiagnosis or under-recognition of neurological impairments in this population. For example, the RPQ asks several questions about emotions, while the NDI does not address this area. Provided the RPQ has a higher overall impairment score when compared to the NDI, using both tools may be beneficial to screen patients for outside referrals. Clinicians should remain vigilant for signs of vestibular, cognitive, and oculomotor dysfunction in patients who have had a whiplash injury, even if there is no reported head trauma.

### 4.1 Limitations

Several limitations should be considered when interpreting these findings. First, the NPC included as a part of the VOMS due to a training error. However, it was deemed not consequential based on the other scores of the VOMS, and it was considered appropriate to retain the current VOMS scoring. Second, the 0.7 effect size for sample size calculation may have been set too high, and thus, this analysis may have been underpowered for between-group comparisons. Based on the results of this study, an effect size of 0.4 would be more appropriate. Third, given the description of PCS, it would have been helpful to have a third arm of participants who were diagnosed with this condition to allow for more comparisons between those with WAD and PCS, but finding an age-sex match with PCS may be unrealistic outside of collecting data at a concussion clinic. Fourth, the groupings of the RightEye® data were based on the categories provided by the company and not on any specific correlations (i.e., r > 0.5). However, provided the variables all examine different components of ocular motion in a specific category (saccades, stability, smooth pursuit, and reaction time), we believe the grouping decisions were appropriate. Finally, the concussion-based tools used within this study were applied outside their typical timeline. However, given that PCS indicates an unresolved concussion and that there are no specific PCS assessment tools, it was appropriate to use these measures within the study. Using a tool such as the Sports Concussion Assessment Tool version 6 may help alleviate some of these issues, but this tool was not published when data collection began.

## 5 Conclusion

This study highlights a significant overlap between symptoms in people with chronic WAD and those commonly seen in people with PCS. Both injuries are caused by rapid acceleration-deceleration forces around the head and neck. They share similar impairments in vestibulo-ocular function, visual tracking, and cognitive processing, with WAD patients exhibiting deficits in each of these domains. The role of cervical spine dysfunction in neurovestibular and ocular symptoms is becoming more evident with current research trends. Emerging evidence suggests the cervical spine and CNS contribute to persistent symptoms in both conditions, including dizziness, headaches, visual disturbances, and cognitive fog. This commonality underscores the need for a comprehensive assessment and targeted treatments. Integrating cervical spine rehabilitation, vestibular therapy, and neurocognitive training may provide best practice evidence for symptom resolution and recovery.

## Data Availability

The original contributions presented in the study are included in the article/Supplementary material, further inquiries can be directed to the corresponding author.
