# Supplementary material for: Subtle ocular motor deficits in people with chronic whiplash associated disorder compared to healthy controls
Source: Front Neurol. 2025 Oct 16;16:1676654. doi: 10.3389/fneur.2025.1676654 (PMC12571609; doi:10.3389/fneur.2025.1676654)
Supplement: Supplementary file 1 [file Data_Sheet_1.pdf]

Appendix 1:

QQ plots for normality of BESS and SAC

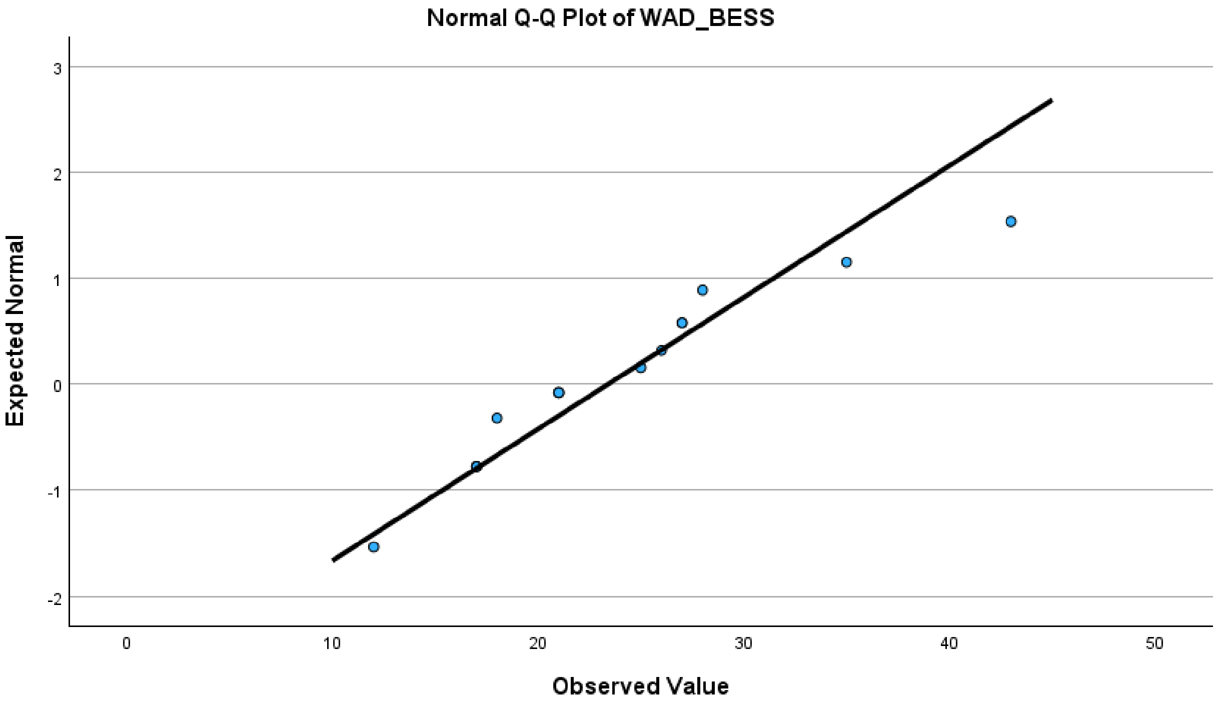

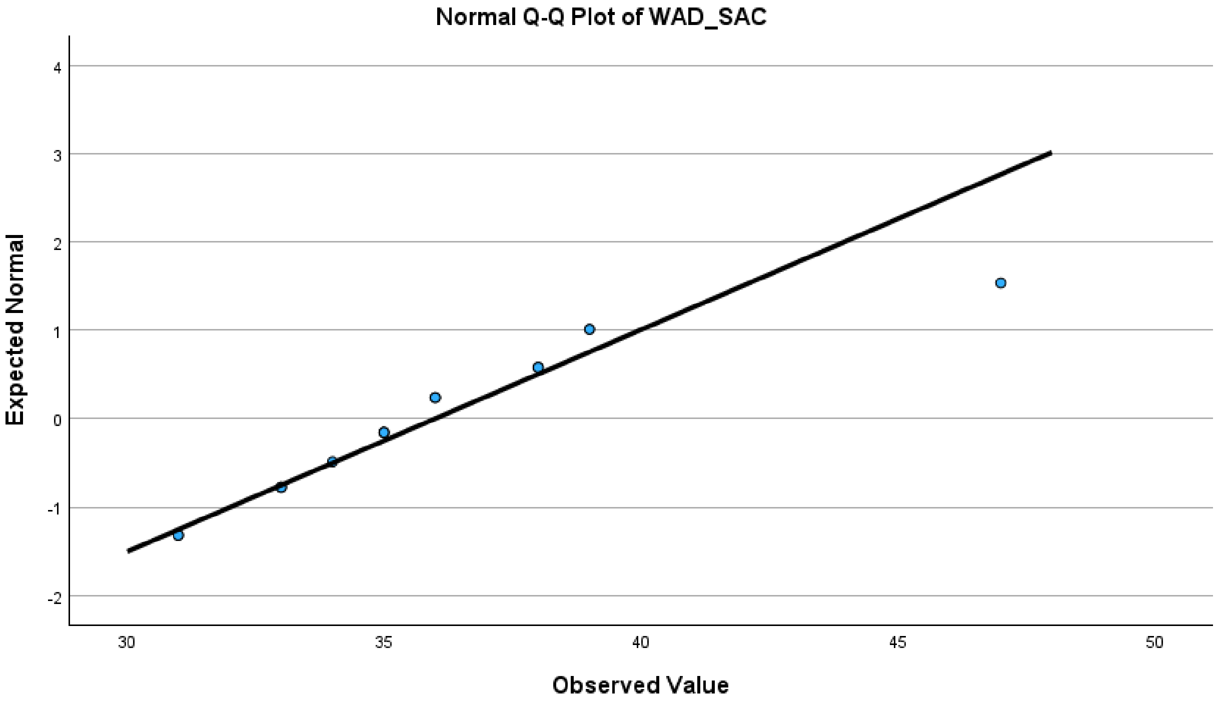

Appendix 2

RightEye® Means, Differences Between Groups, and Effect Sizes

Variable	Group membership	Mean	Mean difference	Matched pairs *t*-test *p*-value.	Wilcoxon Signed Rank test *p*- value	Effect size
CRT processing speed (ms)	WAD	557.27	12.54		0.73	0.091
	Control	544.73				
CRT response accuracy (%)	WAD	77.13	-12.67	0.022*		0.568
	Control	89.80				
CRT saccadic latency average (ms)	WAD	314.13	4.93	0.44		0.043
	Control	309.20				
CRT visual reaction speed (ms)	WAD	431.07	12.87	0.33		0.113
	Control	418.20				
DRT processing speed (ms)	WAD	373.80	16.6	0.37		0.085
	Control	357.20				
DRT reaction time (ms)	WAD	1084.13	27	0.34		0.111
	Control	1057.13				
DRT response accuracy (%)	WAD	85.80	-10.73		0.057	0.509
	Control	96.53				
DRT saccadic latency average (mS)	WAD	312.40	10.4	0.34		0.107
	Control	302.00				
DRT visual reaction speed (ms)	WAD	397.93	0	0.500		0.00
	Control	397.93				
Fixations stability BCEA (pixels squared)	WAD	5.18	0.06		0.31	0.273
	Control	5.12				

Fixation stability convergence point (+/- mm)	WAD	579.31	32.5		0.015*	0.652
	Control	546.81				
Fixation stability depth (+/- mm)	WAD	-10.38	-25.1		0.11	0.425
	Control	14.72				
Hor saccade fixation number L (#)	WAD	23.87	2.6	0.12		0.316
	Control	21.27				
Hor saccade fixation number R (#)	WAD	23.93	2.8	0.10		0.347
	Control	21.13				
Hor saccade missed L (targeting) (#)	WAD	7.13	3.93		0.08	0.474
	Control	3.20				
Hor saccade missed R (targeting) (#)	WAD	6.93	3.4		0.15	0.385
	Control	3.53				
Hor saccade saccadic efficiency L (mm)	WAD	8.46	2.33	0.030*		0.529
	Control	6.13				
Hor saccade saccadic efficiency R (mm)	WAD	8.38	1.39		0.50	.0182
	Control	6.99				
Hor saccade speed-accuracy-tradeoff L (dps/mm)	WAD	4.90	-1.5	0.035*		0.506
	Control	6.40				
Hor saccade speed-accuracy-tradeoff R (dps/mm)	WAD	4.86	-0.72	0.17		0.253
	Control	5.58				
Vert saccade fixation number L (#)	WAD	23.73	3.46	0.027*		0.544
	Control	20.27				

Vert saccade fixation number R (#)	WAD	23.60	3.4	0.026*		0.548
	Control	20.20				
Vert saccade missed L (targeting) (#)	WAD	7.20	4.07	0.018*		0.60
	Control	3.13				
Ver saccade missed R (targeting) (#)	WAD	6.07	2.2	0.132		0.30
	Control	3.87				
Vert saccade saccadic efficiency L (mm)	WAD	7.01	0.92	0.115		0.324
	Control	6.09				
Vert saccade saccadic efficiency R (mm)	WAD	6.56	-0.48		0.78	0.076
	Control	7.04				
Vert saccade speed-accuracy-tradeoff L (dps/mm)	WAD	5.08	-0.45	0.22		0.210
	Control	5.53				
Vert saccade speed-accuracy-tradeoff R (dps/mm)	WAD	5.36	0.2	0.39		0.073
	Control	5.16				
Hor smooth pursuit (%)	WAD	89.09	-1.98	0.21		0.217
	Control	91.07				
Hor smooth pursuit variance (mm)	WAD	19.07	4.45	0.09		0.360
	Control	14.62				
Circular smooth pursuit (%)	WAD	88.60	-1.68	0.13		0.295
	Control	90.28				

Circular smooth pursuit variance (mm)	WAD	19.82	3.47	0.11	[redacted]	0.334
	Control	16.35				
Vert smooth pursuit (%)	WAD	92.87	-0.71	0.37		0.087
	Control	93.58				
Vert smooth pursuit variance (mm)	WAD	24.32	4.16	0.19		0.237
	Control	20.16				

Abbreviations: CRT, choice reaction time; DRT, discriminate reaction time; ms, milliseconds; mm, millimeters; BCEA, bivariate contour eclipse area; Hor, horizontal; Vert, vertical; L, left; R, right; DPS, degrees per second.
